# Joint association of sleep patterns and oxidative balance score with all-cause and cardiovascular mortality among the general population

**DOI:** 10.3389/fnut.2025.1521123

**Published:** 2025-01-29

**Authors:** Chen Chen, Hanzhang Wu, Hongyu Jin, Zhiping Jiang, Wei Wang, Xiao Tan, Wen-Yang Li

**Affiliations:** ^1^Respiratory and Critical Care Department, The First Hospital of China Medical University, Shenyang, China; ^2^Department of Big Data in Health Science, School of Public Health, Zhejiang University School of Medicine, Hangzhou, China; ^3^Department of Psychiatry, Sir Run Run Shaw Hospital, Zhejiang University School of Medicine, Hangzhou, China

**Keywords:** sleep disorder, oxidative balance score, diet, lifestyle, mortality, National Health and Nutrition Examination Survey (NHANES), sleep patterns

## Abstract

**Background:**

This study investigates the joint effect of sleep patterns and oxidative balance score (OBS) on all-cause and CVD mortality in the general population.

**Methods:**

We examined 21,427 individuals aged 18–85 from NHANES 2005–2014, connecting them to mortality data until December 31, 2019, using interview and physical examination dates. Surveys collected data on sleep duration, self-reported sleep disturbance, and doctor-told sleep disorders, classified into healthy, intermediate, and unhealthy sleep patterns. OBS was calculated based on twenty oxidative stress-related exposures to dietary and lifestyle factors. Cox proportional hazards model was conducted to evaluate the association between sleep patterns or OBS alone and combined with all-cause and CVD mortality.

**Results:**

Poor sleep patterns and pro-oxidant OBS (Q1 & Q2) were identified as risk factors for mortality. Each point increase in OBS was associated with a 3% decrease in both all-cause mortality and CVD mortality. There was an interaction between sleep patterns and OBS (P for interaction = 0.013). Joint analyses revealed that participants with combined unhealthy (intermediate and poor) sleep pattern and pro-oxidant OBS were significantly associated with increased risk of all-cause (HR = 1.45 [1.21–1.74]) and CVD mortality (HR = 1.60 [1.12–2.28]). Furthermore, stratified analysis highlighted that this joint effect was more prominent among individuals without hypertension or diabetes; more notable for all-cause mortality in younger individuals and for CVD mortality in the elderly.

**Conclusion:**

We identified a significant interaction between sleep patterns and OBS affecting all-cause mortality. Unhealthy sleep patterns and pro-oxidant OBS were jointly and positively associated with an increased risk of all-cause and CVD mortality. Interventions targeting healthy sleep patterns and antioxidant lifestyles may promote health outcomes.

## 1 Introduction

Sleep is essential for maintaining physiological and psychological homeostasis. Similar to the high prevalence of unhealthy diet and physical inactivity (1, 2), poor sleep is ubiquitous. The Centers for Disease Control and Prevention (CDC) in the United States reported a notable decrease in the average sleep duration of American adults from 1985 to 2012, with around 27.1% of adults experiencing sleep disorders, such as insomnia, sleep apnea, and restless legs syndrome. Cardiovascular disease (CVD) is the leading cause of death in the United States (3), numerous studies have presented the association between abnormal sleep duration, excessive daytime sleepiness and the risk of CVD (4, 5). One suggested to incorporate sleep as a metric of cardiovascular health may enhance the primordial and primary CVD prevention (6). Recently, the American Heart Association (AHA) enhanced their cardiovascular health (CVH) evaluation by introducing the Life's Essential 8 (LE8) score, which integrates sleep health into a new scoring algorithm. Meanwhile, the combined effect of different sleep characteristics, including chronic sleep deprivation, insomnia, and sleep disorders, on CVD risk as well as its mortality and all-cause mortality was also investigated previously (7–9). However, these studies produced inconsistent results due to heterogeneity of age groups, sample sizes, sleep patterns, and interactions between sleep problems and other unhealthy lifestyle factors. Noteworthy, the sleep-related factors are often interrelated and overlapped, it is essential to propose novel evaluation scheme of sleep patterns to comprehensively assess sleep quality and quantity. Besides, when sleep problems coexist with other behaviors, such as smoking, alcohol consumption and physical activity et al., it may further influence the outcome of disease and health (10). Thus, there is a need for a well-designed study to translate a prospective association between comprehensive sleep assessment, together with other unhealthy lifestyle factors and mortality into a measure of its contemporary population healthy level impact.

Unhealthy lifestyle factors may cause oxidative stress, the latter of which is resulting from an imbalance between the production of reactive oxygen species (ROS) and the body's antioxidant defense mechanisms. Oxidative stress has been implicated as a key mechanism underlying various diseases and mortality. The oxidative balance score (OBS), a novel concept derived from multiple dietary (pro-oxidant and antioxidant nutrients) and lifestyle exposures, including smoking, alcohol consumption, obesity, and physical activity, is a useful tool for evaluating an individual's oxidative stress status (11). Previous studies have found that OBS was associated with the development of diseases such as CVD, cancer and neurodegenerative disorders, while the association between OBS with mortality was also elucidated (12). Interestingly, diet and lifestyle has also been found to influence the sleep quality by modulating oxidative balance (13), it is plausible that sleep patterns and OBS may be co-dependent and influence the mortality risk. The comprehensive measurement of pro- and antioxidative diets and lifestyle may facilitate the investigations into the potential risk factors of the CVD and all-cause mortality, as well as to elucidate its joint effects with sleep patterns.

This study aims to investigate whether the joint association exists between sleep patterns and oxidative balance score (OBS) with both all-cause mortality and CVD mortality, as well, whether the concurrent presence of these factors yields more substantial influences on the health outcome than when presented alone. The knowledge generated may offer novel insights into the interventions that target healthy sleep habits and antioxidant-rich diets and lifestyles, which may be important for improving long-term outcomes.

## 2 Methods

### 2.1 Study population

This prospective cohort study used a nationally representative sample from the US National Health and Nutrition Examination Survey (NHANES), which has been conducted on 2-year cycles since 1999 to monitor the health and nutritional status of the US population (14). All the NHANES protocols were approved by the National Center for Health Statistics ethics review board, and written informed consent was obtained from all participants (15). This modeling investigation was exempt from review because it used published, deidentified data sets that included no personally identifiable information (https://www.cdc.gov/nchs/nhanes/index.html).

**Figure 1:**
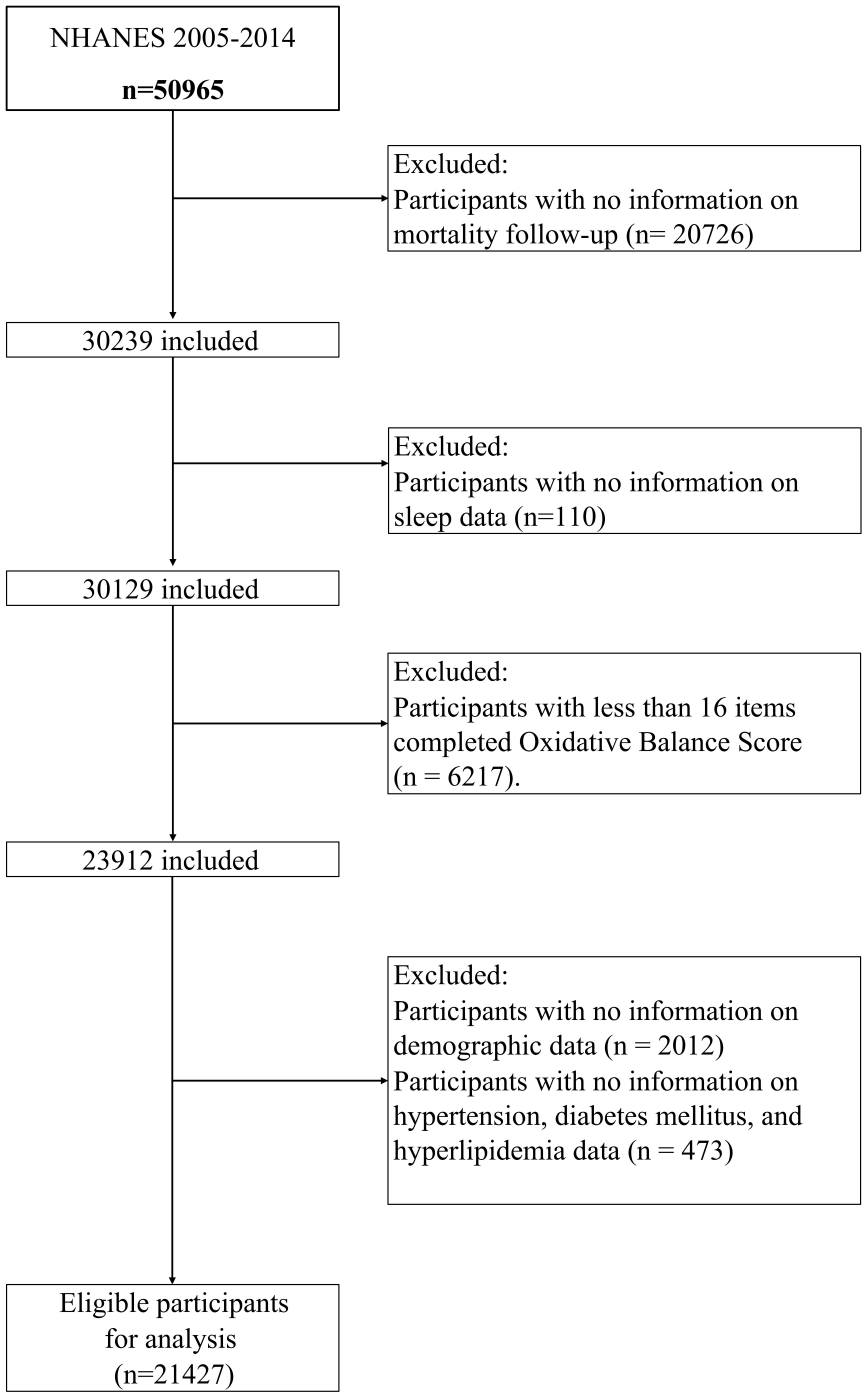
Flowchart of the study design.

Each participant was invited to complete an in-person interview and undergo a set of physical examinations and laboratory tests in a mobile examination center (MEC). This study examined and analyzed data on sociodemographic characteristics, lifestyle factors, and medical history in non-smokers 18 years or older with data available on OBS and self-report sleep questionnaires for 5 cycles of NHANES from 2005 to 2014. Of the 50,965 participants from NHANES 2005–2014, we excluded 20,726 participants without valid death data. We further excluded participants having the following conditions: (1) missing sleep report data (*n* = 110), (2) participants with less than 16 items completed OBS (*n* = 6,217), and (3) missing demographic and medical conditions data (*n* = 2,485). In this study, 21,427 participants with full data were included in the final analysis of this report. The flowchart of participant inclusion is displayed in Figure 1.

### 2.2 Assessment of sleep factors and definition of sleep patterns

Sleep duration was self-reported by the question “How much sleep do you usually get at night on weekdays or workdays?” The quantity of time recorded was grouped as short (<7 h per night), normal (7–9 h per night) and long (>9 h per night), both short and long sleep duration were considered abnormal (16). The responses to “Have you ever told a doctor or other health professional that you have trouble sleeping?” and “Have you/Has standard patients (SP) ever been told by a doctor or other health professional that you have a sleep disorder?” were used to assess the self-reported trouble sleeping and doctor-told sleep disorders, respectively. To generate overall sleep pattern scores, the higher and lower risk factors for sleep behaviors were classified as 1 and 0, respectively. According to sleep factors mentioned above and resulted in scores ranging from 0 to 3, which indicated a healthy (point 0), intermediate (point 1), and poor (points 2–3) sleep pattern (10, 16) (Supplementary Table S1).

### 2.3 Assessment of oxidative balance score

The oxidative balance score (OBS) was computed by evaluating 16 nutrients and 4 lifestyle factors (17, 18), encompassing 5 pro-oxidants and 15 antioxidants, based on established knowledge concerning the association between oxidative stress (OS) and these factors (Supplementary Table S2). Information on dietary intake of 16 nutrients, including dietary fiber, carotene, riboflavin, niacin, vitamin B6, total folate, vitamin B12, vitamin C, vitamin E, calcium, magnesium, zinc, copper, selenium, total fat, and iron. NHANES gathered food intake data through non-consecutive 2-day 24-h dietary recall interviews. The initial dietary recall interview took place in person at the Mobile Examination Center (MEC), while the second interview was conducted over the phone 3 to 10 days later. Daily average intakes were computed from these 2 days of dietary recall data in the final statistical analysis to mitigate bias. Four lifestyle factors considered were physical activity, body mass index (BMI), alcohol consumption, and smoking, with smoking intensity assessed by cotinine levels. Among these factors, total fat, iron, BMI, alcohol consumption, and smoking were classified as pro-oxidants, while the remaining factors were regarded as antioxidants. Following a methodology outlined by Zhang et al. (18), alcohol consumption was categorized into three groups: heavy drinkers (≥15 g/d for women and ≥30 g/d for men), non-heavy drinkers (0–15 g/d for women and 0–30 g/d for men), and non-drinkers, assigned scores of 0, 1, and 2, respectively. Subsequently, the other components were stratified by sex and divided into tertiles, with antioxidants receiving scores of 0–2 in groups 1–3, and pro-oxidants receiving scores of 2–0 in groups 1–3, respectively. Higher OBS scores corresponded to greater exposure to antioxidants. In our study, subjects with complete data for at least 16 out of 20 OBS components were selected. For OBS components with missing data, a score of 0 was assigned, whether it was an antioxidant or pro-oxidant. OBS was evenly divided into four quartiles (Q1, Q2, Q3, and Q4), with the first quartile (Q1) as the reference point. Q1 and Q2 were considered as the pro-oxidative group, while Q3 and Q4 were considered as the antioxidative group according to the previous study.

### 2.4 Ascertainment of mortality

The study examined both all-cause and cardiovascular mortality as primary outcomes. The underlying cause of death was recorded using the International Statistical Classification of Diseases, 10th Revision (ICD-10). All-cause mortality was defined as death from any cause, including cancer (C00–C97), heart disease (I00–I09, I11, I13, I20–I51), cerebrovascular disease (I60-I69), respiratory disease (J10–J18, J40–J47), and other causes. Cardiovascular disease (CVD) mortality specifically encompassed deaths from heart diseases (I00–I09, I11, I13, and I20–I51) and cerebrovascular diseases (I60–I69). Mortality data from the National Health and Nutrition Examination Surveys (NHANES) were linked to death certificate data from the National Death Index (NDI) of the National Center for Health Statistics (NCHS) until December 31, 2019, using a probability matching algorithm. For further information on mortality variables, the following website may be helpful: https://www.cdc.gov/nchs/data-linkage/mortality.htm.

### 2.5 Covariate definitions

In our current study, we considered several demographic variables as covariates, including age, race (Non-Hispanic White, Non-Hispanic Black, and other races), gender (male/female), educational attainment (less than high school, high school or above), and poverty-to-income ratio (<1, ≥1). We also included disease status variables such as hypertension, diabetes mellitus (DM), and hyperlipidemia. Hypertension was defined as a positive response to questions regarding being diagnosed with high blood pressure on multiple occasions or being prescribed medication for hypertension. Additionally, individuals with an average of three consecutive measurements of systolic blood pressure ≥140 mmHg or diastolic blood pressure ≥90 mmHg were also classified as having hypertension. A history of DM was determined through self-reporting of a diagnosis by a healthcare professional or a prescription history for medication used to treat diabetes. Hyperlipidemia was defined based on specific lipid levels, including total cholesterol ≥200 mg/dL, triglycerides ≥150 mg/dL, low-density lipoprotein ≥130 mg/dL, and high-density lipoprotein levels. In addition to these variables, we considered the overall dietary quality using the 2015 version of the Healthy Eating Index (HEI-2015 score) and total energy intake in our study. These variables were included as covariates to account for their potential influence on the outcomes of interest.

### 2.6 Statistical analysis

Data analysis in this study adhered to the analytical guidelines prescribed by the National Center for Health Statistics (NCHS). Individual sample weights were established based on the NHANES recommended sample weight on a dietary day one sample weight (WTDRD2) records considering the complex sampling design of NHANES (i.e., 1/5 ^*^ WTDRD2). The baseline characteristics of participants were categorized by different OBS groups (quartiles), categorical variables are presented as numbers (weighted percentages), and continuous variables are given as weighted means (standard errors). Weighted multivariable Cox regression analysis were applied to evaluate hazard ratios (HR) and 95% CI for the associations of sleep patterns and OBS with all-cause and CVD-specific mortality, respectively. In the crude model, no covariates were adjusted; in model 1, age, gender (male/female), educational attainment (less than high school, and high school or above), and poverty-to-income ratio (poverty income ratio) (<1, ≥1) were adjusted; model 2 was additionally adjusted for hypertension (yes/no), DM (yes/no), hyperlipidemia (yes/no), HEI-2015 score and total energy intake; in model 3, sleep patterns and OBS were mutually adjusted based on model 2. To examine the interaction, sleep patterns were recategorized into a binary variable (unhealthy: poor and intermediate sleep patterns; healthy: healthy sleep pattern) owing to the small case numbers for each group. We conducted evaluations of the impact of OBS status on all-cause and CVD mortality in healthy and unhealthy sleep patterns cohorts, and examining the interaction between these two factors in relation to all-cause and CVD mortality. We further separately analyzed the interactions between the three components that make up the sleep patterns (self-reported sleep issues, doctor-told sleep disorders, and sleep duration) and OBS concerning the outcomes. To examine the joint associations, participants were classified by both sleep patterns (healthy and unhealthy) and OBS status (Q1 and Q2 as the pro-oxidant group, while Q3 and Q4 were considered as the antioxidative group) to estimate mortality risks using multivariable Cox proportional hazards regression models adjusting for the same set of covariates. We also conducted the stratified analysis to determine the joint effect of sleep patterns and OBS status on all-cause and CVD mortality in different groups. To minimize reverse causation risk, we conducted three sensitivity analyses: (1) by excluding deaths that occurred in the initial 2 years of follow-up (21037 included); (2) recalculating the OBS by selecting components of the OBS for which there were 20 complete data (15,426 included); and (3) standardizing the OBS scores and re-transformed them as quartiles. All analyses were performed using R (version 4.2.3, http://www.R-project.org, The R Foundation). *P* < 0.05 was regarded as statistically significant.

## 3 Results

### 3.1 Baseline characteristics

Table 1 depicted the baseline characteristics of individuals, a total of 21,247 adults (weighted population 208,573,393; mean [SE] age, 46.2 [0.3] years) were included. The OBS was grouped into quartile (Q) 1, Q2, Q3, and Q4, with sample sizes of 6,050, 4,734, 5,895, and 4,748, respectively. The range of scores was ≤ 15 in the Q1 group, 16–20 in the Q2 group, 21–26 in the Q3 group, and >26 in the Q4 group. Compared to the lowest OBS quartile, participants in the highest OBS quartile had higher education, higher PIR, higher HEI, higher total energy intake, better sleep pattern, were non-Hispanic White individuals. The difference in sex between OBS groups was not statistically significant. The prevalence of hypertension, diabetes, and hyperlipidemia gradually decreased as OBS increased. Supplementary Table S3 showed the demographic and clinical characteristics of the study population according to sleep patterns. Participants with poor sleep were more likely to be older, female, white participants, had higher BMI and lower OBS.

**Table 1:** The baseline characteristics by quartiles of the OBS: National Health and Nutrition Examination Survey 2005–2014.

**Characteristic**	**Total**	**OBS-Q1**	**OBS-Q2**	**OBS-Q3**	**OBS-Q4**	***P* value**
	***n** =* **21,427**	***n** =* **6,050**	***n** =* **4,734**	***n** =* **5,895**	***n** =* **4,748**	
**Energy intake, kcal/day**	4,220.05 (20.29)	3,019.55 (26.26)	3,860.36 (26.79)	4,447.15 (30.77)	5,362.97 (44.65)	< 0.001^**^
**HEI**	53.59 (0.22)	46.70 (0.30)	51.29 (0.30)	54.57 (0.28)	60.70 (0.36)	< 0.001^**^
**Age group, years**						< 0.001^**^
< 65	16,614 (83.25)	4,454 (80.46)	3,614 (82.40)	4,672 (84.01)	3,874 (85.65)	
≥65	4,813 (16.75)	1,596 (19.54)	1,120 (17.60)	1,223 (15.99)	874 (14.35)	
**Sex**						0.335
Male	10,437 (48.31)	3,048 (48.76)	2,276 (48.91)	2,787 (46.85)	2,326 (49.02)	
Female	10,990 (51.69)	3,002 (51.24)	2,458 (51.09)	3,108 (53.15)	2,422 (50.98)	
**Race**						< 0.001^**^
Other	6,746 (19.16)	1,699 (18.93)	1,494 (19.86)	1,968 (19.66)	1,585 (18.26)	
Non-Hispanic Black	4,625 (11.25)	1,845 (18.31)	1,063 (12.33)	1,070 (9.24)	647 (6.11)	
Non-Hispanic White	10,056 (69.59)	2,506 (62.76)	2,177 (67.81)	2,857 (71.10)	2,516 (75.63)	
**PIR**						< 0.001^**^
< 1	4,589 (14.69)	1,659 (21.40)	1,034 (14.87)	1,126 (12.64)	770 (10.65)	
≥1	16,838 (85.31)	4,391 (78.60)	3,700 (85.13)	4,769 (87.36)	3,978 (89.35)	
**Educational attainment**						< 0.001^**^
< High school	5,302 (16.57)	2,024 (25.21)	1,285 (18.26)	1,252 (14.41)	741 (9.66)	
≥High school	16,125 (83.43)	4,026 (74.80)	3,449 (81.74)	4,643 (85.59)	4,007 (90.34)	
**BMI**						< 0.001^**^
< 30	13,514 (64.75)	3,363 (56.21)	2,855 (61.59)	3,772 (64.38)	3,524 (75.56)	
≥30	7,913 (35.26)	2,687 (43.79)	1,879 (38.41)	2,123 (35.62)	1,224 (24.45)	
**Sleep patterns**						< 0.001^**^
Healthy	10,110 (48.37)	2,594 (42.41)	2,169 (46.45)	2,887 (50.27)	2,460 (53.31)	
Intermediate	7,699 (34.90)	2,252 (36.55)	1,726 (36.36)	2,107 (34.10)	1,614 (33.08)	
Poor	3,618 (16.73)	1,204 (21.04)	839 (17.21)	901 (15.63)	674 (13.60)	
**Hypertension**						< 0.001^**^
No	12,803 (63.95)	3,235 (58.89)	2,743 (61.94)	3,627 (63.56)	3,198 (70.66)	
Yes	8,624 (36.05)	2,815 (41.11)	1,991 (38.06)	2,268 (36.44)	1,550 (29.34)	
**DM**						< 0.001^**^
No	18,794 (91.04)	5,101 (88.42)	4,087 (90.31)	5,228 (90.90)	4,378 (94.21)	
Yes	2,633 (8.95)	949 (11.58)	647 (9.69)	667 (9.10)	370 (5.79)	
**Hyperlipidemia**						< 0.001^**^
No	6,711 (31.17)	1,766 (29.03)	1,392 (29.43)	1,821 (29.32)	173 (36.61)	
Yes	14,716 (68.83)	4,284 (70.97)	3,342 (70.57)	4,074 (70.68)	3,016 (63.39)	

^*^P < 0.05, ^**^P < 0.01. OBS, oxidative balance score; HEI, Healthy Eating Index; BMI, body mass index; PIR, poverty income ratio; DM, diabetes mellitus. Q1: OBS ≤ 15; Q2: 15 < CDAI ≤ 20; Q3: 20 < CDAI ≤ 26; Q4: 26 < CDAI ≤ 37. Weighted to be nationally representative, categorical variables are presented as numbers (weighted %) and continuous variables are given as weighted means (standard errors).

### 3.2 Association of single sleep patterns and OBS with all-cause and CVD mortality

During the follow-up period of up to 15 years (median, 9.4 years), a total of 2,743 all-cause deaths (13.7/1,000 person-years) and 835 cardiovascular deaths (4.2/1,000 person-years) were recorded. Participants with poor sleep pattern had increased all-cause and CVD-specific mortality risks (Table 2). After adjusting for covariates, HRs for all-cause and CVD-specific mortality among individuals with a poor sleep pattern compared with those with a healthy sleep pattern were 1.40 [1.21–1.63] and 1.48 [1.14–1.92] (All *P* values < 0.05). Meanwhile, an inverse association was observed between OBS and the risk of all-cause mortality (Q4 vs. Q1: HR = 0.61 [0.46–0.80], *P* < 0.001, *P* for trend = 0.002) and CVD mortality (Q4 vs. Q1: HR = 0.58 [0.37–0.90], *P* = 0.016, *P* for trend = 0.033). The probability of all-cause and CVD both decreased by 3% for each unit increased in OBS.

**Table 2:** Association of single sleep patterns and OBS with all-cause and CVD mortality.

**Mortality outcome**	**Death/No**.	**Weighted death (%)**	**Hazard ratio (95%CI)**, ***P*** **value**							
			**Crude model**		**Model 1**		**Model 2**		**Model 3**	***P*** **value**
**All-cause**										
**Sleep patterns**										
Healthy	1,207/10,110	8,806,379 (8.7)	Reference		Reference		Reference		Reference	
Intermediate	928/7,699	6,606,899 (9.1)	1.06 (0.94, 1.19)	0.371	1.16 (1.03, 1.31)	0.013^*^	1.12 (1.00, 1.26)	0.053	1.12 (0.99, 1.26)	0.068
Poor	608/3,618	4,825,687 (13.8)	1.69 (1.48, 1.93)	< 0.001^**^	1.52 (1.310, 1.77)	< 0.001^**^	1.40 (1.21, 1.63)	< 0.001^**^	1.40 (1.20, 1.63)	< 0.001^**^
**OBS**										
Q1	1,037/6,050	6,821,369 (13.7)	Reference		Reference		Reference		Reference	
Q2	651/4,734	4,626,146 (10.5)	0.77 (0.67, 0.89)	< 0.001^**^	0.84 (0.71, 0.98)	0.031^*^	0.84 (0.70, 1.02)	0.084	0.84 (0.69, 1.02)	0.080
Q3	677/5,895	5,513,619 (9.2)	0.67 (0.58, 0.77)	< 0.001^**^	0.78 (0.68, 0.90)	< 0.001^**^	0.81(0.66,0.99)	0.048^*^	0.82 (0.67, 1.01)	0.066
Q4	378/4,748	3,277,832 (6.0)	0.44 (0.37, 0.53)	< 0.001^**^	0.56 (0.46, 0.67)	< 0.001^**^	0.61 (0.46, 0.80)	< 0.001^**^	0.61 (0.47, 0.81)	< 0.001^**^
P for trend				< 0.001^**^		< 0.001^**^		0.002^**^		0.002^**^
**OBS per 1 point increase**			0.96 (0.95, 0.97)	< 0.001^**^	0.97 (0.96, 0.98)	< 0.001^**^	0.97 (0.95, 0.98)	< 0.001^**^	0.97 (0.95, 0.98)	< 0.001^**^
**CVD**										
**Sleep patterns**										
Healthy	375/10,110	2,611,733 (2.6)	Reference		Reference		Reference		Reference	
Intermediate	274/7,699	1,854,221 (2.6)	1.00 (0.81, 1.23)	0.990	1.15 (0.94, 1.41)	0.174	1.11 (0.91, 1.36)	0.294	1.11 (0.91, 1.36)	0.294
Poor	186/3,618	1,444,938 (4.1)	1.74 (1.37, 2.22)	< 0.001^**^	1.63 (1.26, 2.11)	< 0.001^**^	1.48 (1.14,1.92)	0.003^**^	1.47 (1.13, 1.92)	0.004^**^
**OBS**										
Q1	314/6,050	1,924,870 (3.9)	Reference		Reference		Reference		Reference	
Q2	211/4,734	1,548,306 (3.5)	0.90 (0.66, 1.23)	0.505	0.96 (0.70, 1.32)	0.817	0.99 (0.69, 1.41)	0.946	0.99 (0.69, 1.42)	0.941
Q3	206/5,895	1,597,340 (2.7)	0.68 (0.52, 0.89)	0.005^**^	0.79 (0.56, 1.03)	0.084	0.85 (0.57, 1.26)	0.408	0.86 (0.58, 1.28)	0.461
Q4	104/4,748	840,376 (1.6)	0.39 (0.28, 0.54)	< 0.001^**^	0.49 (0.36, 0.67)	< 0.001^**^	0.58 (0.37, 0.90)	0.016^*^	0.58 (0.37, 0.91)	0.018^*^
P for trend				< 0.001^**^		< 0.001^**^		0.033^*^		0.038^*^
**OBS per 1 point increase**			0.95 (0.94, 0.97)	< 0.001^**^	0.96 (0.95, 0.98)	< 0.001^**^	0.97 (0.94, 0.99)	0.005^**^	0.97 (0.94, 0.99)	0.006^**^

^*^P < 0.05, ^**^P < 0.01. Crude model: Unadjusted model. Model 1: Adjusted for age, sex (male, female), race (other, non-Hispanic Black, non-Hispanic White), educational level (< high school, ≥high school), and PIR (< 1, ≥1). Model 2: Additionally adjusted for total energy intake (kcal/day), HEI, hypertension (yes/no), DM (yes/no), hyperlipidemia (yes/no). Model 3: sleep patterns and OBS were mutually adjusted based on model 2. OBS, oxidative balance score; CVD, cardiovascular disease; PIR, poverty income ratio; HEI, healthy eating index; DM, diabetes mellitus.

### 3.3 Interaction and joint analysis of sleep patterns and OBS with mortality

**Figure 2:**
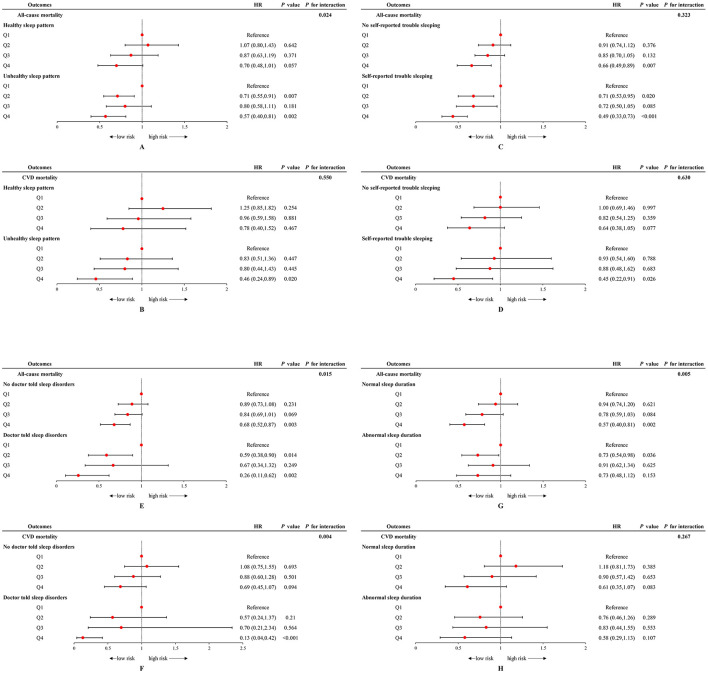
Forest plot of stratified analyses of the associations of OBS status with all-cause and CVD mortality among US adults in different sleep cohorts. **(A, B)** Associations of OBS status with all-cause and CVD mortality among US adults in different sleep patterns groups. **(C, D)** Associations of OBS status with all-cause and CVD mortality among US adults in with and without self-reported trouble sleeping groups. **(E, F)** Associations of OBS status with all-cause and CVD mortality among US adults in with and without doctor-told sleep disorders groups. **(G, H)** Associations of OBS status with all-cause and CVD mortality among US adults in normal and abnormal sleep duration groups. Data were depicted as the weighted hazard ratio (HR) with 95% confidence interval. The model was adjusted for age, sex, race, educational level, PIR, total energy intake, HEI, hypertension, DM, and hyperlipidemia. OBS was recognized as the reference point. OBS, oxidative balance score; CVD, cardiovascular disease; PIR, poverty income ratio; HEI, healthy eating index; DM, diabetes mellitus.

There was a significant interaction between sleep patterns and OBS status regarding the impact on all-cause mortality (*P* for interaction = 0.024) but not on CVD mortality (Figures 2A, B). We found that participants adopting a higher OBS (anti-oxidant) had a lower all-cause mortality risk (Q4 vs. Q1: HR = 0.57 [0.40–0.81], *P* = 0.002) and CVD mortality risk (Q4 vs. Q1: HR = 0.46 [0.24–0.89], *P* = 0.020) in the unhealthy sleep pattern cohort. However, this association was not observed among individuals with healthy sleep pattern. For all-cause mortality, the HR comparing the highest to the lowest OBS quartile was 0.70 (95% CI: 0.48–1.01), with a *P*-value of 0.057, indicating no significant difference. Similarly, for CVD mortality risk, the HR was 0.78 (95% CI: 0.40–1.52), with a *P*-value of 0.467, also suggesting no significant association.

When examining the effects of each specific sleep behaviors and OBS on mortality, it is notable that the highest quartile of OBS can reduce mortality risk in those with no self-reported trouble sleeping/no doctor-told sleep disorders, while the protective effect of higher OBS is more pronounced in individuals with sleep complaints. Participants with sleep complaints, including self-reported trouble sleeping and doctor-told sleep disorders, demonstrated a lower risk of all-cause mortality (trouble sleeping: HR = 0.49 [0.33–0.73]; sleep disorders: HR = 0.26 [0.11–0.62]) and CVD mortality (trouble sleeping: HR = 0.45 [0.22–0.91]; sleep disorders: HR = 0.13 [0.04–0.42]) when adhering to the highest level of OBS (Q4). A significant interaction was observed between doctor-told sleep disorders and OBS status regarding all-cause mortality (*P* for interaction = 0.015) and CVD mortality (*P* for interaction = 0.004) (Figures 2C–F). An interaction was also noted between sleep duration and OBS concerning all-cause mortality (P for interaction = 0.005), with the protective effect of Q4 level of OBS evident only in individuals with normal sleep durations (Figures 2G, H).

**Figure 3:**
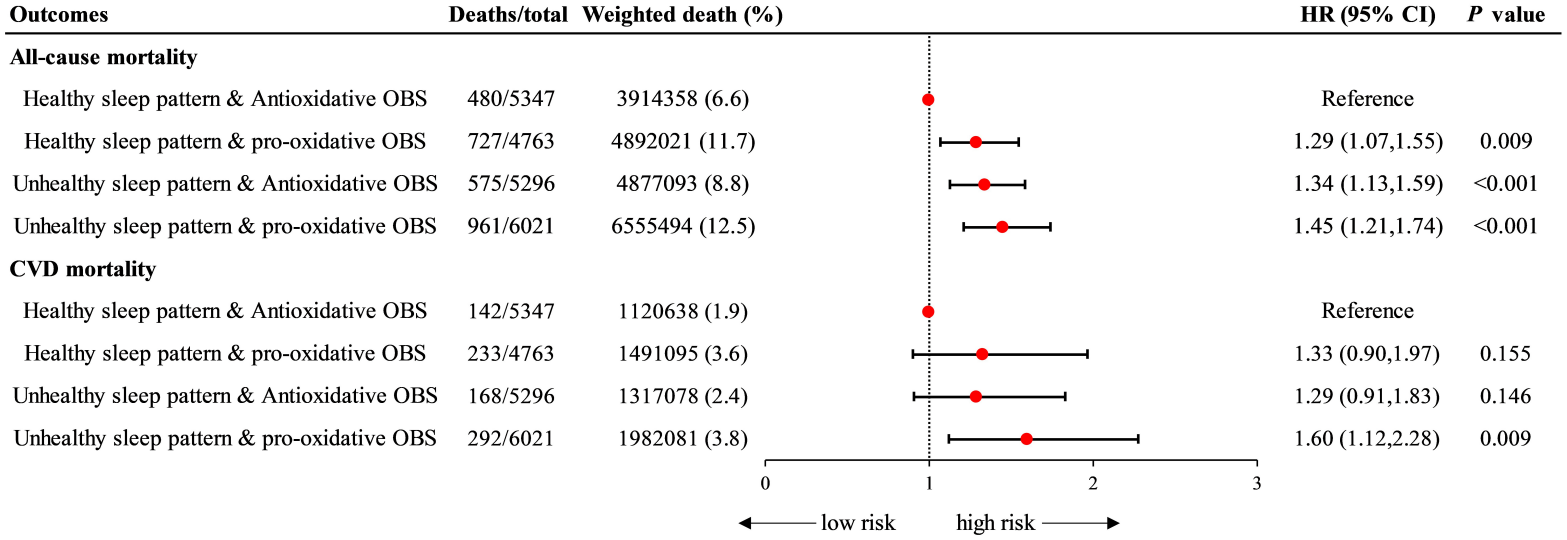
Joint association of sleep patterns and OBS with all-cause and CVD mortality. Data were depicted as the weighted hazard ratio (HR) with 95% confidence interval. The model was adjusted for age, sex, race, educational level, PIR, total energy intake, HEI, hypertension, DM, and hyperlipidemia. Halthy sleep pattern & Anti-oxidative group was recognized as the reference point. OBS, oxidative balance score; CVD, cardiovascular disease; PIR, poverty income ratio; HEI, healthy eating index; DM, diabetes mellitus.

In joint analyses, participants with unhealthy sleep pattern and pro-oxidant OBS were associated with a highest risk of all-cause mortality (HR = 1.45 [1.21–1.74], *P* < 0.001) and CVD mortality (HR = 1.60 [1.12–2.28], *P* = 0.009) compared with those who were healthy sleep pattern and anti- oxidant OBS (Figure 3, Supplementary Table S4).

### 3.4 Subgroup and sensitivity analysis between combined sleep patterns with OBS and mortality risk

In the subgroup analysis (Tables 3, 4), we found that among participants without hypertension, those who combined unhealthy sleep patterns and pro-oxidant OBS had the highest risk of all-cause mortality (HR = 1.56 [1.28–1.89], *P* < 0.05) and CVD mortality (HR = 1.55 [1.07–2.23], *P* < 0.05), compared to those with healthy sleep patterns and antioxidative OBS. The analysis also revealed a statistically significant interaction for CVD mortality (*P* for interaction = 0.010), but not for all-cause mortality (*P* for interaction = 0.487).

**Table 3:** Subgroups analyses for the association between the combined sleep patterns with OBS and risk of all-cause mortality.

**Characteristic**	**Healthy sleep and antioxidative OBS**	**Healthy sleep and pro-oxidative OBS**	**Unhealthy sleep and antioxidative OBS**	**Unhealthy sleep and pro-oxidative OBS**	**P for interaction**
**Age group**					< 0.001^**^
< 65	1 [Reference]	1.80 (1.21, 2.68)	2.01 (1.46, 2.78)	1.80 (1.24, 2.61)	
≥65	1 [Reference]	1.14 (0.92, 1.41)	1.10 (0.93, 1.30)	1.36 (1.14, 1.63)	
**Sex**					0.629
Male	1 [Reference]	1.32 (1.00, 1.75)	1.43 (1.15, 1.78)	1.56 (1.20, 2.02)	
Female	1 [Reference]	1.23 (0.93, 1.62)	1.25 (0.98, 1.60)	1.33 (1.03, 1.72)	
**Race**					0.049^*^
Other	1 [Reference]	1.05 (0.58, 1.92)	1.01 (0.59, 1.71)	1.28 (0.70, 2.35)	
Non-Hispanic Black	1 [Reference]	0.88 (0.59, 1.31)	1.22 (0.83, 1.79)	0.89 (0.65, 1.21)	
Non-Hispanic White	1 [Reference]	1.34 (1.07, 1.67)	1.37 (1.13, 1.66)	1.52 (1.22, 1.89)	
**PIR**					0.010^*^
< 1	1 [Reference]	1.01 (0.60, 1.70)	1.08 (0.70, 1.66)	1.09 (0.66, 1.79)	
≥1	1 [Reference]	1.32 (1.08, 1.62)	1.39 (1.12, 1.71)	1.51 (1.26, 1.81)	
**Educational attainment**					0.680
< High school	1 [Reference]	1.15 (0.83, 1.60)	1.36 (0.99, 1.86)	1.33 (1.00, 1.77)	
≥High school	1 [Reference]	1.35 (1.08, 1.69)	1.35 (1.11, 1.65)	1.50 (1.20, 1.89)	
**Hypertension**					0.487
No	1 [Reference]	1.31 (1.04, 1.65)	1.31 (1.07, 1.61)	1.56 (1.28, 1.89)	
Yes	1 [Reference]	1.21 (0.89, 1.64)	1.38 (1.03, 1.87)	1.20 (0.87, 1.64)	
**DM**					0.205
No	1 [Reference]	1.24 (1.01, 1.52)	1.30 (1.07, 1.58)	1.50 (1.21, 1.88)	
Yes	1 [Reference]	1.42 (0.90, 2.25)	1.43 (0.95, 2.14)	1.35 (0.91, 1.98)	
**Hyperlipidemia**					0.982
No	1 [Reference]	1.15 (0.73, 1.82)	1.37 (0.94, 2.00)	1.36 (0.88, 2.10)	
Yes	1 [Reference]	1.31 (1.08, 1.58)	1.33 (1.09, 1.63)	1.48 (1.22, 1.80)	

Adjusted for age, sex, race, educational level, PIR, total energy intake, HEI, hypertension, DM, and hyperlipidemia. ^*^P < 0.05, ^**^P < 0.01. OBS, oxidative balance score; CVD, cardiovascular disease; PIR, poverty income ratio; HEI, healthy eating index; DM, diabetes mellitus.

**Table 4:** Subgroups analyses for the association between the combined sleep patterns with OBS and risk of CVD mortality.

**Characteristic**	**Healthy sleep and antioxidative OBS**	**Healthy sleep and pro-oxidative OBS**	**Unhealthy sleep and antioxidative OBS**	**Unhealthy sleep and pro-oxidative OBS**	**P for interaction**
**Age group**					0.005^**^
< 65	1 [Reference]	2.93 (1.03, 8.34)	2.38 (0.99, 5.71)	2.26 (0.86, 5.98)	
≥65	1 [Reference]	1.11 (0.74, 1.67)	1.11 (0.78, 1.57)	1.54 (1.03, 2.31)	
**Sex**					0.231
Male	1 [Reference]	1.94 (1.24, 3.03)	1.64 (1.01, 2.64)	2.26 (1.48, 3.44)	
Female	1 [Reference]	0.86 (0.49, 1.53)	1.05 (0.68, 1.63)	1.09 (0.66, 1.79)	
**Race**					0.799
Other	1 [Reference]	0.98 (0.34, 2.80)	0.88 (0.26, 2.93)	1.11 (0.39, 3.19)	
Non-Hispanic Black	1 [Reference]	1.21 (0.54, 2.69)	1.56 (0.68, 3.55)	1.16 (0.67, 1.99)	
Non-Hispanic White	1 [Reference]	1.38 (0.89, 2.16)	1.33 (0.90, 1.96)	1.72 (1.13, 2.61)	
**PIR**					0.051
< 1	1 [Reference]	0.64 (0.21, 1.98)	0.82 (0.38, 1.78)	0.82 (0.31, 2.19)	
≥1	1 [Reference]	1.46 (0.96, 2.23)	1.40 (0.94, 2.10)	1.80 (1.21, 2.68)	
**Educational attainment**					0.001^*^
< High school	1 [Reference]	0.82 (0.49, 1.35)	1.45 (0.86, 2.45)	1.05 (0.61, 1.82)	
≥High school	1 [Reference]	1.60 (1.02, 2.52)	1.27 (0.86, 1.89)	1.90 (1.24, 2.90)	
**Hypertension**					0.010^**^
No	1 [Reference]	1.17 (0.77, 1.78)	1.21 (0.85, 1.73)	1.55 (1.07, 2.23)	
Yes	1 [Reference]	2.01 (0.95, 4.27)	1.61 (0.82, 3.13)	1.73 (0.74, 4.05)	
**DM**					0.434
No	1 [Reference]	1.55 (0.98, 2.45)	1.30 (0.89, 1.91)	1.83 (1.13, 2.96)	
Yes	1 [Reference]	0.85 (0.42, 1.69)	1.16 (0.60, 2.24)	1.08 (0.57, 2.05)	
**Hyperlipidemia**					0.117
No	1 [Reference]	2.72 (1.08, 6.89)	1.68 (0.73, 3.89)	1.99 (0.92, 4.32)	
Yes	1 [Reference]	1.15 (0.76, 1.73)	1.24 (0.86, 1.79)	1.55 (1.02, 2.37)	

Adjusted for age, sex, race, educational level, PIR, total energy intake, HEI, hypertension, DM, and hyperlipidemia. OBS, oxidative balance score; CVD, cardiovascular disease; PIR, poverty income ratio; HEI, healthy eating index; DM, diabetes mellitus.

Similarly, among participants without diabetes, the combination of unhealthy sleep patterns and pro-oxidant OBS was associated with the highest risk of all-cause mortality (HR = 1.50 [1.21–1.88], *P* < 0.05) and CVD mortality (HR = 1.83 [1.13–2.96], *P* < 0.05), compared to those with healthy sleep patterns and antioxidative OBS. However, there was no statistically significant interaction. Additionally, this combined effect was more significant in terms of all-cause mortality in young individuals (HR = 1.80 [1.24–2.61], *P* for interaction < 0.001) and CVD mortality in the elderly (HR = 1.54 [1.03 HR = 2.31], *P* for interaction < 0.005).

Sensitivity analysis was conducted to evaluate the robustness of joint associations, it remained similar after excluding deaths that occurred during the first 2-year follow-up (Supplementary Table S5). By excluding participants with incomplete OBS components and recalculating the OBS, the joint association remains stable in all-cause mortality but diminishes in the context of CVD mortality (Supplementary Table S6). By standardizing the OBS scores and re-transformed them as quartiles, the joint associations remained stable (Supplementary Table S7).

## 4 Discussion

In this nationwide prospective cohort study utilizing NHANES data, we examined 21,427 adults and conducted a clinical follow-up for a 15-year period. For the first time, we observed that participants with combined sleep patterns interacted with OBS, independently or jointly, affected overall and CVD mortality, there was also an interaction between sleep patterns and OBS status on mortality. Participants with combined unhealthy sleep patterns and pro-oxidant OBS were significantly associated with increased risk of all-cause and CVD mortality, this correlation was more prominent in those without hypertension or diabetes mellitus. Additionally, this combined effect was more significant in terms of all-cause mortality in young individuals and CVD mortality in the elderly.

Numerous studies have investigated the influence of different aspects of sleep behaviors on CVD risk and its outcome. Some cross-sectional studies and meta–analysis demonstrated that both short and long sleep duration were associated with an increased risk of all–cause mortality and cardiovascular events (19), however, others did not find consistent results (20, 21). Self-reported trouble sleeping and doctor-told sleep disorders were also found to be associated with higher all-cause and CVD mortality (22). Our study expanded on previous research by highlighting the importance of considering combined sleep characteristics rather than focusing on a single sleep trait when examining the association with mortality. In this study, poor sleep patterns were independently associated with increased risks of all-cause mortality (HR = 1.40) and cardiovascular disease mortality (HR = 1.48) after adjusting for confounding factors. The potential mechanisms may be related with the influenced endocrine and metabolic system, which may cause the elevated mortality risk (23–25). In addition to the arteriolosclerosis caused by sleep fragmentation (26), poor sleep pattern was also linked with fatigue and lethargy, which in turn may lead to inadequate recovery from stress and illness then led to increased mortality (27). Further experimental studies are needed to explore the potential impact of sleep pattern on health outcomes in future.

Oxidative stress was recognized as a pivotal factor in the pathogenesis of numerous diseases and mortality. There was an increasing interest in the health implications of overall antioxidant capacity. The composite dietary antioxidant index score (28), developed by previous researchers, quantifies an individual's antioxidant capacity using six dietary nutrients including vitamins A, C, E, carotenoids, zinc, and selenium—which modulate the oxidant/antioxidant balance and mitigate oxidative stress, thereby reducing risks of cardiovascular disease, and mortality (29, 30). Furthermore, the LE8 score offers a holistic evaluation of cardiovascular health, encompassing various dietary and lifestyle factors, was proved to be have an inverse association with mortality (31). However, it is important to acknowledge that our diet comprises both antioxidant and pro-oxidant nutrients. The OBS uniquely assesses antioxidant and pro-oxidant exposure by including 14 antioxidant nutrients, 2 pro-oxidant nutrients, and 4 lifestyle factors, providing a comprehensive view of the body's antioxidant defense system (32). This made OBS superior to other dietary antioxidant scores in reflecting the full spectrum of antioxidant capacity. In two prospective cohort studies with median follow-ups of over 10 years, participants in the higher OBS group (indicating a more anti-oxidative state) presented a lower risk of all-cause and CVD mortality (33, 34). Our findings consistently support a significant inverse association between OBS and the risk of all-cause and CVD mortality. Individuals with a higher OBS (more anti-oxidative state) had a lower risk of death from all-cause and CVD-related events. These results support the notion that maintaining optimal oxidative balance through a combination of favorable dietary and lifestyle factors may have protective effects not only against oxidative stress, but also against mortality (35–37).

Aside from the independent effects of both sleep and OBS on mortality, the primary finding of our study demonstrated that adults coexist with both unhealthy sleep pattern and pro-oxidative OBS exhibiting the highest risk of all-cause and CVD mortality, with a robust sample size. A study involving 10,212 individuals also demonstrated a significantly higher risk of angina and congestive heart failure in individuals who have both unhealthy sleep patterns and a low OBS (10). This research provided empirical evidence supporting the influential role of sleep and OBS on health outcomes. Building upon these findings, our study went a step further, delving into the effects of sleep and OBS on all-cause and CVD mortality-the adverse outcomes. And for the first time, we observed significant interactions between doctor-told sleep disorders and OBS status with respect to all-cause mortality and CVD mortality. Additionally, interactions were noted between sleep patterns, sleep duration and OBS concerning all-cause mortality or CVD mortality. These findings revealed a substantial modification in the correlation between sleep parameters and mortality by OBS, indicating a more pronounced reduction in the risk of all-cause and CVD mortality among individuals with sleep problems who had higher OBS levels. However, for participants with a healthy sleep (no doctor-told sleep disorders, and no self-reported trouble sleeping), this correlation was only obvious in the highest levels of OBS The findings underscore the significance of adhering to anti-oxidants (higher OBS) among individuals with sleep problems, as it plays a crucial role in mitigating all-cause mortality risk and thereby contributes to the prevention of premature mortality. Therefore, integrating sleep patterns and OBS as essential health metrics, similar to other health behaviors, has the potential to strengthen primary disease prevention initiatives, reduce mortality risks and advance health outcomes.

It is significant to note that potential biological pathways such as oxidative stress, inflammation, DNA damage, and impaired cell function provide valuable insights into how all-cause mortality might be influenced by the interaction between pro- and anti-oxidative states (as indicated by OBS levels) and unhealthy sleep habits. However, the specific mechanisms at play in these interactions remain largely unexplored. In comparison to those without sleep problems, individuals with sleep problems exhibit heightened levels of oxidative stress, may lead to unhealthy dietary habits and obesity, such as increased eating frequency and altered meal timing (38, 39), this further exacerbates oxidative stress, ultimately potentially contributing to a higher mortality rate in this population. However, in the population who already enjoy healthy sleep pattern, only at the highest levels of OBS that a significant impact on all-cause and CVD mortality risk was observed; while in the population with an unhealthy sleep, a more pronounced reduction in mortality risk could be found. This implied that adopting an antioxidative OBS did not have a substantial impact on all-cause mortality risk in participants with a healthy sleep. In contrast to individuals with sleep problems, this difference may be primarily due to the inherent lower oxidative stress burden in individuals with healthy sleep (40, 41), possibly coupled with healthier lifestyles and fewer comorbidities, which could diminish the full effectiveness of an antioxidant-OBS. A study investigating the influence of the dietary OBS (DOBS) on mortality among individuals with and without CVD, revealing that adherence to DOBS only reduced death risk among those with CVD but had no e?ect on those without CVD (32). The findings align with our own conclusion, suggesting that anti-oxidative interventions targeting non-specific populations may have limited efficacy in mitigating mortality risk. The aforementioned findings underscore the critical importance and significant clinical benefits of OBS interventions in alleviating oxidative stress, particularly among individuals experiencing sleep problems. When unhealthy sleep patterns coexist with a low OBS, psuch as prolonged sleep duration, may reduce the available time for physical activity. Furthermore, excessive smoking and alcohol consumption can disrupt the normal sleep rhythm (42, 43), further exacerbating the impact of sleep disturbances on health. Another study showed that the probability of sleep disorder decreased by 40% for each unit increased in OBS, indicating the higher the OBS, the fewer sleep problems. We assume that pro-oxidants may exacerbate sleep quality when both factors present simultaneously, and consequently increase the susceptibility to diseases and the risk of mortality (13). In summary, the interplay between these two factors may synergistically amplify oxidative stress, leading to cellular damage, inflammation, and a heightened risk of mortality. This combined effect also weakens immune function, increasing vulnerability to infections and chronic diseases. The inflammatory imbalances induced by unhealthy sleep habits and low OBS levels can further exacerbate the progression of cardiovascular diseases and raise the risks associated with mortality (10). Moreover, these factors disrupt metabolic regulation, promoting insulin resistance and dyslipidemia, result in obesity. Further research is warranted to better understand these complex interactions and develop targeted interventions addressing sleep patterns and oxidative balance for improved health outcomes.

These results emphasize the importance of adopting a diet and lifestyle rich in antioxidants for reducing the risk of mortality, this will still benefit even in the presence of poor sleep quality.

Interestingly, the association of combined unhealthy sleep and pro-oxidative OBS on mortality were observed across different age groups, particularly among the younger population (<65 years, HR = 1.80 [1.24–2.61]) instead the older for all-cause mortality. This result was consistent with a prospective cohort study conducted in Sweden, who also found that the impact of short and long sleep duration on mortality was more pronounced among younger individuals and decreased with increasing age (44). There is no definitive explanation for these phenomena. One possible reason could be that older individuals constitute a survivor population that might exhibit heightened resilience to adverse health consequences. Additionally, the effects of retirement on the accuracy of sleep duration estimates could be another factor to consider. The absence of a fixed schedule to wake up at a specific time for work could lead to a lack of a crucial time reference for individuals. Moreover, among those older individuals, the association of combined sleep and OBS effect was more prominently observed with CVD-related mortality. This is as expected, since sub-health status or uncontrolled chronic diseases were uncommonly seen in younger individuals, but might be prominent confounders for the association between sleep pattern and mortality among older individuals.

In this study, stratified analyses indicated that the positive association between the combined unhealthy sleep patterns and pro-oxidant OBS with all-cause mortality was more pronounced among those without hypertension or DM at baseline. This suggests that interventions targeting optimal dietary and lifestyle behaviors may be more effective in preventing mortality before the onset of hypertension or DM. Hypertension and DM are well-established risk factors for mortality, leading to mortality through a complex interplay of pathophysiological mechanisms. These include endothelial dysfunction, vascular remodeling, oxidative stress, glycolipid metabolism, inflammation, atherosclerosis, myocardial infarction, stroke, and end-organ damage, ultimately resulting in fatal consequences (45–47). Previous studies have also shown that poor sleep or pro-oxidant dietary and lifestyle behaviors, including sleep apnea, short sleep duration, unhealthy diet (low in fiber and high in meat), and low physical activity may lead to all-cause and CVD death caused by abnormal glycolipid metabolism, endothelial dysfunction, oxidative stress, and inflammation (48–50). Therefore, sleep behaviors, OBS, and hypertension or DM may have a synergistic effect on death through common pathways, which has also been indicated in our findings that the interaction correlation was found between the combined unhealthy sleep patterns, pro-oxidant OBS, and hypertension.

The strengths of this study lie in its novel approach, large sample size, and longitudinal design, which allowed for robust statistical analysis and examination of temporal relationships. Additionally, the adjustment for potential confounding factors enhances the validity of the observed associations. The clinical implications of this research emphasize the importance of addressing both sleep patterns and oxidative balance to reduce mortality risks. However, limitations of the study should be acknowledged, Firstly, due to the absence of data, we were unable to include more sleep information, such as the OSA and insomnia; secondly, the assessment of sleep patterns and their elements relied on interviews and surveys rather than objective methods like polysomnography and actigraphy. This approach could lead to inaccuracies stemming from reporting discrepancies or memory bias. Finally, the study collected dietary information solely at the baseline, without any subsequent monitoring, which might not accurately capture the daily variations in diet. The reliance on self-reported 24-h dietary recalls could lead to recall bias or misclassification of dietary intake, participants may not be able to accurately remember or report their actual food consumption, which could introduce inaccuracies in the assessment of the OBS.

## 5 Conclusion

We identified a significant interaction between sleep patterns and OBS affecting all-cause mortality. Unhealthy sleep patterns and pro-oxidant OBS were jointly and positively associated with an increased risk of all-cause and CVD mortality. Interventions targeting healthy sleep patterns and antioxidant lifestyles may promote health outcomes.

## Data Availability

Publicly available datasets were analyzed in this study. This data can be found here: https://www.cdc.gov/nchs/nhanes.
