# Supplementary material for: Joint association of sleep patterns and oxidative balance score with all-cause and cardiovascular mortality among the general population
Source: Front Nutr. 2025 Jan 29;12:1521123. doi: 10.3389/fnut.2025.1521123 (PMC11816669; doi:10.3389/fnut.2025.1521123)
Supplement: Supplementary file 1 [file Table_1.docx]

Supplementary Material

# Supplementary Tables

**Table S1. Assessment of sleep scores**

| **Individual components** | **Sleep scores** |
| --- | --- |
| Doctor-told sleep disorder |  |
| No | 1 |
| Yes | 0 |
| Self-reported trouble sleeping |  |
| No | 1 |
| Yes | 0 |
| Sleep duration |  |
| <7h | 1 |
| 7-9h | 0 |
| >9h | 1 |

**Table S2: Components of the oxidative balance score (Zhang et al., 2022).**

| OBS components | Property | Male | | | Female | | | |
| --- | --- | --- | --- | --- | --- | --- | --- | --- |
|  |  | 0 | 1 | 2 | 0 | 1 | 2 | |
| **Dietary OBS components** |  |  |  |  |  |  |  |  |
| Dietary fiber (g/d) | A | <12.56 | 12.56-19.70 | ≥19.70 | <10.10 | 10.10-16.31 | ≥16.31 | |
| Carotene (RE/d) | A | <98.83 | 98.83-306.25 | ≥306.25 | <98.08 | 98.08-383.50 | ≥383.50 | |
| Riboflavin (mg/d) | A | <1.79 | 1.79-2.69 | ≥2.69 | <1.34 | 1.34-2.02 | ≥2.02 | |
| Niacin (mg/d) | A | <20.65 | 20.65-29.75 | ≥29.75 | <14.52 | 14.52-21.86 | ≥21.86 | |
| Vitamin B_6_ (mg/d) | A | <1.59 | 1.59-2.40 | ≥2.40 | <1.13 | 1.13-1.77 | ≥1.77 | |
| Total folate (mcg/d) | A | <316.00 | 316.00-492.00 | ≥492.00 | <251.00 | 251.00-388.96 | ≥388.96 | |
| Vitamin B_12_ (mcg/d) | A | <3.36 | 3.36-6.20 | ≥6.20 | <2.22 | 2.22-4.22 | ≥4.22 | |
| Vitamin C (mg/d) | A | <42.44 | 42.44-113.21 | ≥113.21 | <38.01 | 38.01-98.49 | ≥98.49 | |
| Vitamin E (ATE) (mg/d) | A | <5.82 | 5.82-9.42 | ≥9.42 | <4.53 | 4.53-7.52 | ≥7.52 | |
| Calcium (mg/d) | A | <646.00 | 646.00-1072.00 | ≥1072.00 | <499.24 | 499.24-849.00 | ≥849.00 | |
| Magnesium (mg/d) | A | <257.00 | 257.00-361.28 | ≥361.28 | <187.00 | 187.00-283.43 | ≥283.43 | |
| Zinc (mg/d) | A | <9.75 | 9.75-15.10 | ≥15.10 | <6.73 | 6.73-10.75 | ≥10.75 | |
| Copper (mg/d) | A | <1.12 | 1.12-1.57 | ≥1.57 | <0.85 | 0.85-1.28 | ≥1.28 | |
| Selenium (mcg/d) | A | <94.94 | 94.94-141.80 | ≥141.80 | <67.79 | 67.79-99.50 | ≥99.50 | |
| Total fat (g/d) | P | >107.43 | 69.83-107.43 | <69.83 | >75.79 | 50.98-75.79 | <50.98 | |
| Iron (mg/d) | P | >19.17 | 12.88-19.17 | <12.88 | >14.32 | 9.65-14.32 | <9.65 | |
| **Lifestyle OBS components** |  |  |  |  |  |  |  | |
| Physical activity (MET-minute/week) | A | <417.86 | 417.86-1135.71 | ≥1135.71 | <270.00 | 270.00-845.71 | ≥845.71 | |
| Alcohol (g/d) | P | ≥30 | 0-30 | None | ≥15 | 0-15 | None | |
| Body mass index (kg/m^2^) | P | >29.17 | 25.54-29.17 | <25.54 | >28.64 | 23.74-28.64 | <23.74 | |
| Cotinine (ng/mL) | P | >1.13 | 0.038-1.13 | <0.038 | >0.172 | 0.035-0.172 | <0.035 | |

**Notes:** The antioxidants were assigned fractions from 0 to 2. Conversely, the prooxidants were assigned the opposite fraction.

**Abbreviations**: OBS: oxidative balance score; A: antioxidant; P: prooxidant; RE: retinol equivalent; ATE: alpha-tocopherol equivalent; MET: metabolic equivalent.

**Table S3. Baseline characteristics according to sleep patterns.**

| **Characteristic** | **No. of participants by sleep pattern (weighted %)** | | | | **P value** |
| --- | --- | --- | --- | --- | --- |
|  | **All (n=21427)** | **Healthy (n=10110)** | **Intermediate (n=7699)** | **Poor (n=3618)** |  |
| **Age, years, mean (SE)** | 46.16 (0.31) | 45.32 (0.40) | 45.47 (0.36) | 50.03 (0.33) | <0.001^**^ |
| **Energy intake (kcal/day)** | 4220.05 (20.29) | 4233.58 (22.80) | 4292.68 (30.41) | 4029.37 (42.61) | <0.001^**^ |
| **HEI score** | 53.59 (0.22) | 54.62 (0.27) | 52.66 (0.26) | 52.56 (0.36) | <0.001^**^ |
| **Age group, years** |  |  |  |  | 0.001^**^ |
| <65 | 16614 (83.25) | 7785 (82.56) | 6105 (84.87) | 2724 (81.85) |  |
| ≥65 | 4813 (16.75) | 2325 (17.44) | 1594 (15.13) | 894 (18.15) |  |
| **Sex** |  |  |  |  | <0.001^**^ |
| Male | 10437 (48.31) | 5069 (49.38) | 3798 (49.08) | 1570 (43.59) |  |
| Female | 10990 (51.69) | 5041 (50.62) | 3901 (50.92) | 2048 (56.41) |  |
| **Race** |  |  |  |  | <0.001^**^ |
| Other | 6746 (19.16) | 3531 (20.50) | 2355 (19.72) | 860 (14.15) |  |
| Non-Hispanic Black | 4625 (11.25) | 1759 (8.57) | 2021 (14.22) | 845 (12.78) |  |
| Non-Hispanic White | 10056 (69.59) | 4820 (70.93) | 3323 (66.06) | 1913 (73.07) |  |
| **PIR** |  |  |  |  | <0.001^**^ |
| <1 | 4589 (14.69) | 2053 (13.27) | 1634 (15.16) | 902 (17.81) |  |
| ≥1 | 16838 (85.31) | 8057 (86.73) | 6065 (84.84) | 2716 (82.19) |  |
| **Educational attainment** |  |  |  |  | 0.337 |
| < High school | 5302 (16.57) | 2561 (16.30) | 1884 (17.21) | 857 (16.03) |  |
| ≥ High school | 16125 (83.43) | 7549 (83.70) | 5815 (82.79) | 2761 (83.97) |  |
| **BMI** |  |  |  |  | < 0.001^**^ |
| <30 | 13514 (64.74) | 6854 (69.23) | 4870 (64.58) | 1790 (52.10) |  |
| ≥30 | 7913 (35.26) | 3256 (30.77) | 2829 (35.42) | 1828 (47.90) |  |
| **OBS** |  |  |  |  | <0.001^**^ |
| Q1 | 6050 (23.89) | 2594 (20.95) | 2252 (25.03) | 1204 (30.05) |  |
| Q2 | 4734 (21.23) | 2169 (20.38) | 1726 (22.11) | 839 (21.83) |  |
| Q3 | 5895 (28.83) | 2887 (29.97) | 2107 (28.17) | 901 (26.93) |  |
| Q4 | 4748 (26.05) | 2460 (28.71) | 1614 (24.69) | 674 (21.18) |  |
| **Hypertension** |  |  |  |  | <0.001^**^ |
| No | 12803 (63.95) | 6554 (68.69) | 4672 (65.01) | 1577 (48.04) |  |
| Yes | 8624 (36.05) | 3556 (31.31) | 3027 (34.99) | 2041 (51.96) |  |
| **DM** |  |  |  |  | <0.001^**^ |
| No | 18794 (91.05) | 9112 (93.01) | 6825(91.63) | 2857 (84.16) |  |
| Yes | 2633 (8.95) | 998 (6.99) | 874(8.37) | 761 (15.84) |  |
| **Hyperlipidemia** |  |  |  |  | <0.001^**^ |
| No | 6711 (31.17) | 3324 (33.10) | 2523(31.48) | 864 (24.95) |  |
| Yes | 14716 (68.83) | 6786 (66.90) | 5176(68.52) | 2754 (75.05) |  |

**Notes**: Weighted to be nationally representative, categorical variables are presented as numbers (weighted %) and continuous variables are given as weighted means (standard errors). * P<0.05, ** P<0.01

**Abbreviations:** HEI, Healthy Eating Index; OBS: oxidative balance score; BMI, body mass index; PIR, poverty income ratio; DM, diabetes mellitus;

**Table S4. Joint association of sleep patterns and OBS with all-cause and CVD mortality.**

| **Mortality outcome** | **OBS** | **Deaths/total** | **Weighted death (%)** | **Hazard ratio (95%CI), P value** | | | | | |  |
| --- | --- | --- | --- | --- | --- | --- | --- | --- | --- | --- |
|  |  |  |  | **Crude model** | **P** | **MV model 1** | **P** | **MV model 2** | **P** | |
| **All causes** |  |  |  |  |  |  |  |  |  | |
| Healthy sleep pattern | antioxidative OBS | 480/5347 | 3914358 (6.6) | reference |  | reference |  | reference |  | |
|  | pro-oxidative OBS | 727/4763 | 4892021 (11.7) | 1.78 (1.50,2.12) | <0.001^**^ | 1.48 (1.24,1.76) | <0.001^**^ | 1.29 (1.07,1.55) | 0.009^**^ | |
| Unhealthy sleep pattern | antioxidative OBS | 575/5296 | 4877093 (8.8) | 1.38 (1.14,1.63) | <0.001^**^ | 1.40 (1.18,1.67) | <0.001^**^ | 1.34 (1.13,1.59) | <0.001^**^ | |
|  | pro-oxidative OBS | 961/6021 | 6555494 (12.5) | 1.95 (1.70,2.24) | <0.001^**^ | 1.75 (1.51,2.01) | <0.001^**^ | 1.45 (1.21,1.74) | <0.001^**^ | |
| **CVD** |  |  |  |  |  |  |  |  |  | |
| Healthy sleep pattern | antioxidative OBS | 142/5347 | 1120638 (1.9) | reference |  | reference |  | reference |  | |
|  | pro-oxidative OBS | 233/4763 | 1491095 (3.6) | 1.94 (1.45,2.60) | <0.001^**^ | 1.55 (1.15,2.10) | 0.005^**^ | 1.33 (0.90,1.97) | 0.155 | |
| Unhealthy sleep pattern | antioxidative OBS | 168/5296 | 1317078 (2.4) | 1.31 (0.91,1.89) | 0.145 | 1.36 (0.95,1.93) | 0.089 | 1.29 (0.91,1.83) | 0.146 | |
|  | pro-oxidative OBS | 292/6021 | 1982081 (3.8) | 2.11 (1.66,2.67) | <0.001^**^ | 1.97 (1.54,2.51) | <0.001^**^ | 1.60 (1.12,2.28) | 0.009^**^ | |

**Notes:** * P<0.05, ** P<0.01

Crude model: Unadjusted model; Model 1: Adjusted for age, sex (male, female), race (other, non-Hispanic Black, non-Hispanic White), educational level (<high school, ≥high school), and PIR (<1, ≥1); Model 2: Additionally adjusted for total energy intake (kcal/day), HEI, hypertension (yes/no), DM (yes/no), and hyperlipidemia (yes/no).

**Abbreviations:** OBS, oxidative balance score; CVD, cardiovascular disease; PIR, poverty income ratio; HEI, healthy eating index; DM, diabetes mellitus.

**Table S5. Joint association of sleep patterns and OBS with all-cause and CVD mortality after excluding participants died within 2 years of follow-up (n= 21,037).**

| **Mortality outcome** | **OBS** | **Deaths/total** | **Weighted death (%)** | **Hazard ratio (95%CI), P value** | | | | | |
| --- | --- | --- | --- | --- | --- | --- | --- | --- | --- |
|  |  |  |  | **Crude model** | **P** | **MV model 1** | **P** | **MV model 2** | **P** |
| **All causes** |  |  |  |  |  |  |  |  |  |
| Healthy sleep pattern | antioxidative OBS | 430/5397 | 3579396 (6.1) | reference |  | reference |  | reference |  |
|  | pro-oxidative OBS | 633/4669 | 4355098 (10.6) | 1.74 (1.45,2.08) | <0.001^**^ | 1.45 (1.20,1.74) | <0.001^**^ | 1.30 (1.06,1.59) | 0.010^*^ |
| Unhealthy sleep pattern | antioxidative OBS | 500/5221 | 4245704 (7.8) | 1.32 (1.09,1.61) | 0.005^**^ | 1.34 (1.13,1.60) | 0.001^**^ | 1.28 (1.08,1.52) | 0.006^**^ |
|  | pro-oxidative OBS | 790/5850 | 5430027(10.6) | 1.77 (1.53,2.05) | <0.001^**^ | 1.61 (1.38,1.88) | <0.001^**^ | 1.37 (1.13,1.67) | 0.002^**^ |
| **CVD** |  |  |  |  |  |  |  |  |  |
| Healthy sleep pattern | antioxidative OBS | 128/5397 | 1049637 (1.8) | reference |  | reference |  | reference |  |
|  | pro-oxidative OBS | 204/4669 | 1325107 (3.2) | 1.84 (1.35,2.50) | <0.001^**^ | 1.48 (1.07,2.03) | 0.017^*^ | 1.33 (0.87,2.01) | 0.187 |
| Unhealthy sleep pattern | antioxidative OBS | 147/5221 | 1158825 (2.1) | 1.24 (0.84,1.82) | 0.282 | 1.28 (0.88,1.86) | 0.200 | 1.22 (0.84,1.76) | 0.295 |
|  | pro-oxidative OBS | 247/5850 | 1696346 (3.3) | 1.93 (1.49,2.50) | <0.001^**^ | 1.82 (1.38,2.39) | <0.001^**^ | 1.55 (1.03,2.34) | 0.037^*^ |

**Notes:** * P<0.05, ** P<0.01

Crude model: Unadjusted model; Model 1: Adjusted for age, sex (male, female), race (other, non-Hispanic Black, non-Hispanic White), educational level (<high school, ≥high school), and PIR (<1, ≥1); Model 2: Additionally adjusted for energy intake (kcal/day), HEI, hypertension (yes/no), DM (yes/no), hyperlipidemia (yes/no).

**Abbreviations**: OBS, oxidative balance score; CVD, cardiovascular disease; PIR, poverty income ratio; HEI, healthy eating index; DM, diabetes mellitus.

**Table S6. Joint association of sleep patterns and OBS with all-cause and CVD mortality after recalculating the OBS by excluding participants with missing** **components of OBS (n=15,426).**

| **Mortality outcome** | **OBS** | **Deaths/total** | **Weighted death (%)** | **Hazard ratio (95%CI), P value** | | | | | | |
| --- | --- | --- | --- | --- | --- | --- | --- | --- | --- | --- |
|  |  |  |  | **Crude model** | **P** | **MV model 1** | **P** | **MV model 2** | **P** |  |
| **All causes** |  |  |  |  |  |  |  |  |  |  |
| Healthy sleep pattern | antioxidative OBS | 420/4328 | 2588105 (5.2) | reference |  | reference |  | reference |  |  |
|  | pro-oxidative OBS | 356/3138 | 2752562 (9.2) | 1.76 (1.40,2.21) | <0.001^**^ | 1.59 (1.27,2.01) | <0.001^**^ | 1.49 (1.16,1.93) | 0.002^**^ |  |
| Unhealthy sleep pattern | antioxidative OBS | 338/4140 | 2936209 (6.6) | 1.30 (1.03,1.64) | 0.028^*^ | 1.35 (1.10,1.65) | 0.005^**^ | 1.30 (1.05,1.61) | 0.015^*^ |  |
|  | pro-oxidative OBS | 412/3820 | 3049511 (8.7) | 1.69(1.38,2.06) | <0.001^**^ | 1.60 (1.29,1.98) | <0.001^**^ | 1.43 (1.09,1.89) | 0.010^*^ |  |
| **CVD** |  |  |  |  |  |  |  |  |  |  |
| Healthy sleep pattern | antioxidative OBS | 96/4328 | 701484 (1.4) | reference |  | reference |  | reference |  |  |
|  | pro-oxidative OBS | 115/3138 | 828291 (2.8) | 1.97 (1.34,2.91) | <0.001^**^ | 1.80 (1.18,2.76) | 0.007^**^ | 1.61 (0.96,2.72) | 0.072 |  |
| Unhealthy sleep pattern | antioxidative OBS | 93/4140 | 716079 (1.6) | 1.18 (0.76,1.82) | 0.463 | 1.28 (0.86,1.92) | 0.230 | 1.24 (0.83,1.86) | 0.296 |  |
|  | pro-oxidative OBS | 123/3820 | 855069 (2.4) | 1.78 (1.25,2.53) | 0.001^**^ | 1.82 (1.24,2.66) | 0.002^**^ | 1.57 (0.94,2.63) | 0.085 |  |

**Notes**: * P<0.05, ** P<0.01

Crude model: Unadjusted model; Model 1: Adjusted for age, sex (male, female), race (other, non-Hispanic Black, non-Hispanic White), educational level (<high school, ≥high school), and PIR (<1, ≥1); Model 2: Additionally adjusted for energy intake (kcal/day), HEI, hypertension (yes/no), DM (yes/no), hyperlipidemia (yes/no).

**Abbreviations**: OBS, oxidative balance score; CVD, cardiovascular disease; PIR, poverty income ratio; HEI, healthy eating index; DM, diabetes mellitus.

**Table S7.** **Joint association of sleep patterns and OBS with all-cause and CVD mortality after** **recalculating the OBS by standardizing the OBS scores**

| **Mortality outcome** | **OBS** | **Deaths/total** | **Weighted death (%)** | **Hazard ratio (95%CI), P value** | | | | | |
| --- | --- | --- | --- | --- | --- | --- | --- | --- | --- |
|  |  |  |  | **Crude model** | **P** | **MV model 1** | **P** | **MV model 2** | **P** |
| **All causes** |  |  |  |  |  |  |  |  |  |
| Healthy sleep pattern | antioxidative OBS | 541/5821 | 4428556 (6.9) | reference |  | reference |  | reference |  |
|  | pro-oxidative OBS | 666/4289 | 4377820 (11.9) | 1.73 (1.46,2.06) | <0.001^**^ | 1.42 (1.18,1.70) | <0.001^**^ | 1.24 (1.02,1.50) | 0.027^**^ |
| Unhealthy sleep pattern | antioxidative OBS | 636/5835 | 5295855 (8.8) | 1.31 (1.10,1.57) | 0.003^**^ | 1.33 (1.12,1.57) | 0.001^**^ | 1.26 (1.06,1.49) | 0.008^**^ |
|  | pro-oxidative OBS | 900/5482 | 6136732 (13.0) | 1.94 (1.69,2.22) | <0.001^**^ | 1.74(1.51,2.00) | <0.001^**^ | 1.47(1.24,1.74) | <0.001^**^ |
| **CVD** |  |  |  |  |  |  |  |  |  |
| Healthy sleep pattern | antioxidative OBS | 166/5821 | 1321637 (2.1) | reference |  | reference |  | reference |  |
|  | pro-oxidative OBS | 209/4289 | 1290096 (3.5) | 1.75 (1.34,2.29) | <0.001^**^ | 1.36 (1.01,1.84) | 0.047^*^ | 1.14 (0.79,1.65) | 0.494 |
| Unhealthy sleep pattern | antioxidative OBS | 188/5835 | 1493616 (2.5) | 1.25 (0.90,1.73) | 0.184 | 1.27 (0.92,1.77) | 0.146 | 1.21 (0.88,1.66) | 0.246 |
|  | pro-oxidative OBS | 272/5482 | 1805543 (3.8) | 1.96 (1.56,2.47) | <0.001^**^ | 1.83 (1.42,2.37) | <0.001^**^ | 1.46 (1.02,2.09) | 0.039^*^ |

**Notes**: * P<0.05, ** P<0.01

Crude model: Unadjusted model; Model 1: Adjusted for age, sex (male, female), race (other, non-Hispanic Black, non-Hispanic White), educational level (<high school, ≥high school), and PIR (<1, ≥1); Model 2: Additionally adjusted for energy intake (kcal/day), HEI, hypertension (yes/no), DM (yes/no), hyperlipidemia (yes/no).

**Abbreviations**: OBS, oxidative balance score; CVD, cardiovascular disease; PIR, poverty income ratio; HEI, healthy eating index; DM, diabetes mellitus
